# Stress-induced developmental plasticity and spontaneous preterm birth: A justice-oriented eco-evo-devo review

**DOI:** 10.1016/j.eurox.2025.100409

**Published:** 2025-06-08

**Authors:** Gabriella Mayne, Luwam Ghidei, Ayisha Buckley, Wei Perng, K. Joseph Hurt, David P. Tracer

**Affiliations:** a Department of Health & Behavioral Sciences, University of Colorado, Denver, CO, USA; b Department of Obstetrics and Gynecology, Wake Forest University, Winston Salem, NC 2820, USA; c Department of Obstetrics and Gynecology and Department of Maternal Fetal Medicine, Weill Cornell Medicine New York, New York, USA; d Lifecourse Epidemiology of Adiposity and Diabetes Center, University of Colorado Anschutz Medical Campus, Aurora, CO, USA; e Division of Reproductive Sciences and Division of Maternal Fetal Medicine, Department of Obstetrics and Gynecology, University of Colorado Anschutz Medical Campus, Aurora, CO, USA

**Keywords:** Corticotropin-releasing hormone (CRH), Cortisol, Enriched environment, Maternal mental health, Neuroendocrine, Obstetric racism

## Abstract

Spontaneous preterm birth (<37 weeks’ gestation) is a leading cause of neonatal morbidity and mortality, with little global progress in prevention. Spontaneous preterm birth disproportionately affects communities marginalized by racism and socio-economic disadvantage. Maternal stress is a well-established risk factor for spontaneous preterm birth and is more prevalent in marginalized communities. Yet, maternal stress remains underutilized as a target for clinical intervention. In this review, we draw from ecological, evolutionary, and developmental (eco-evo-devo) biology, Black feminist theory, and reproductive justice to center the margins of those communities most burdened by both maternal stress and preterm birth. In doing so, we re-frame the importance of maternal stress mitigation in spontaneous preterm prevention. Through the lens of stress-induced developmental plasticity, environmental stressors may shorten gestation through evolutionarily conserved maternal-fetal-placental signaling pathways. Two features of this process are particularly relevant to clinical care: first, stress may impact gestational length in a dose-dependent manner; second, its effects may be reversible. Reducing maternal stress may be a highly feasible clinical opportunity to tangibly reduce spontaneous preterm birth and increase birth equity.

## Highlights

• Maternal stress is a modifiable risk factor for spontaneous preterm birth.
• Communities margianalized by racism and socioeconomic disadvantage have higher stress and higher rates of preterm birth.
• Stress-induced developmental plasticity can shorten gestation, but stress effects may be dose-responsive and reversible.
• Thus, clinicians can reframe maternal stress mitigation as a key clinical opportunity to reduce preterm birth risk.
• Safe, respectful care aimed at mitigating maternal stress may lower preterm birth risk and advance birth equity.

## Introduction

### Mapping the Margins of Spontaneous Preterm Birth

Globally, eight countries contribute more than half of all spontaneous preterm births worldwide: India, Pakistan, Nigeria, China, Ethiopia, Bangladesh, Democratic Republic of Congo, and the United States [1]. Six of those eight countries have a history of colonization, imperialism, and/or slavery. While demographic factors, such as population density, clearly play a role in the global distribution of preterm birth, this shared history of inequities is norrt incidental. Countries with histories of colonization, imperialism, and slavery – including the U.S., U.K., Canada, and Australia – consistently show persistent racial and ethnic disparities in maternal and infant health, especially preterm birth [2], [3], [4], [5]. In the U.S., Black and Indigenous individuals have the highest preterm birth rates (14.6 % and 12.3 %, respectively), about 50 % higher than non-Hispanic Whites [2]. Similar disparities exist in the U.K. (Afro-Caribbean: 10.2 % vs. 7.6 %) [3], Canada (Inuit regions: 12.4 % vs. 7.8 %) [6], and Australia (First Nations: 12.6 % vs. 7.6 %) [7]. Once attributed to individual or genetic factors, these disparities are increasingly recognized as rooted in structural and social determinants of health [8], [9], [10].

Decades of literature describe the association between maternal stress and spontaneous preterm birth [11], [12], [13], [14], [15], [16], [17], [18], [19], [20], [21], [22], [23], [24], [25], [26], [27], [28], [29], [30], [31], [32], [33], [34]. Many studies also acknowledge that racialized social inequity is associated with preterm birth [35], [36], [37], [38], [39], [40], [41], [42], [43], [44], [45], [46], [47]. Despite this, the higher stress in marginalized communities as an etiology for increased risk for preterm birth has not been meaningfully applied in clinical settings, though stress physiology likely contributes to outcomes for all birthing people.

To better understand and respond to this relationship, we draw from Black feminist theory. Black feminist theory centers the birthing person’s lived experience, acknowledges intersecting socio-political and historical context shaping health patterns, and recognizes the resilience of pregnant and birthing people in marginalized communities [48], [49], [50]. *Mapping the margins* [51] of preterm birth means deliberately focusing on those populations most burdened. In doing so, we may bring stress and its clinical implications into clearer view [36], [52].

In parallel to Black feminist theory, we incorporate insights from eco-evo-devo biology, a framework that synthesizes ecological, evolutionary, and developmental sciences to explain how environmental conditions – like chronic stress – shape phenotypic outcomes [53], [54], [55]. From this perspective, spontaneous preterm birth may reflect a biologically conserved, stress-sensitive response to adverse environments [53], [54], [55], [56], [57], [58], [59], [60]. Eco-evo-devo elucidates how early-life environments shape developmental trajectories in ways that are both evolutionarily conserved and context-sensitive. By incorporating eco-evo-devo biology, this review offers conceptual tools that complement and deepen our understanding of human health by situating it within broader ecological and evolutionary systems.

**Fig. 1:**
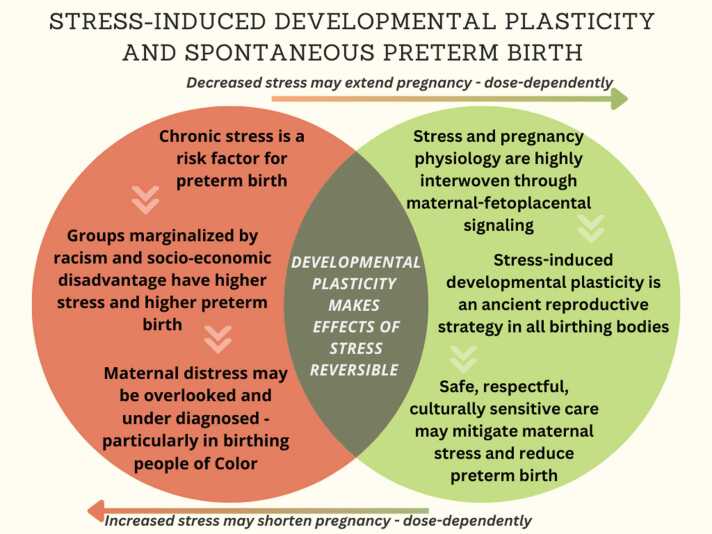
Infographic summarizing the evidence reviewed demonstrating maternal stress as a critical clinical opportunity. Stress-induced developmental plasticity contributes to our understanding of spontaneous preterm birth and highlights two key themes: stress effects are dose-dependent and potentially reversible. Adapted from [68].

Finally, a reproductive justice-oriented lens further grounds our approach in the socio-political, historical, and economic realities that shape health inequities (reviewed in [61]). Reproductive justice not only affirms the right to bodily autonomy and safe parenting environments, but it also demands that we center the experiences of communities most impacted by systemic inequities in maternal health – particularly Black, Indigenous, and other historically marginalized populations [62], [63], [64], [65]. By merging these frameworks – eco-evo-devo biology, Black feminist theory, and reproductive justice – we aim to reframe maternal stress as both biologically conserved signaling as well as a clinical opportunity. Mitigating stress in pregnancy, particularly among marginalized populations, may be a critical and underutilized pathway to reduce spontaneous preterm birth and advance birth equity [50], [66], [67]. Fig. 1 summarizes key elements of our review findings.

## Maternal stress and distress

From a biological perspective, stress is simply “a state in which homeostasis is actually threatened or perceived to be so; homeostasis is re-established by a complex repertoire of behavioral and physiological adaptive responses of the organism” (p.374 [69]). Maternal stress is stress occurring during pregnancy [70].

**Fig. 2:**
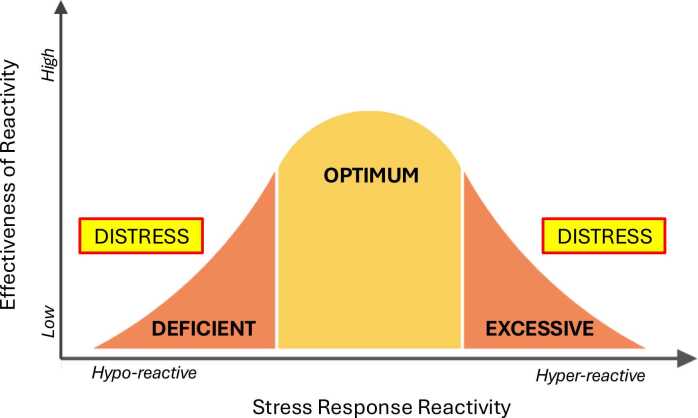
Visualization of the stress response on a bell curve. Optimal reactivity lies at the center of the curve, where responses are most effective. Distress arises at both extremes—hypo- or hyper-reactivity—where the effectiveness of the reactivity is diminished. Adapted from [69].

Homeostatic systems like the human stress response can be visualized as a bell curve, with optimal function at the center and suboptimal responses at either extreme [69]. Distress arises when a stressor overwhelms the system’s capacity to return to baseline, resulting in either an insufficient or excessive response – that is, a hypo- or hyper-reactive stress response [69]), Fig. 2.

### Anxiety and depression as indicators of maternal distress

Maternal stress may occur through a wide array of *structural stressors*, many of which have been directly linked to preterm birth, including poverty [71], natural disasters [26], social vulnerability [72], [73], neighborhood violence [74], air pollution and heat exposure [75], explicitly racist policies such as racial segregation [76], and redlining [77], eviction [78], inter-personal violence [79], adverse childhood experiences [80], food deserts [81], and obstetric deserts [82], [83]. Maternal psychosocial stressors include experiences of racism, discrimination, and chronic stress [84], [85]. High levels of structural and psychosocial stress may manifest as depression, anxiety, and perceived stress [86], [87]. Depression, anxiety, and perceived stress are associated with increased risk of preterm birth, even when controlling for confounding biomedical factors such as diabetes and hypertension [34], [88], [89]. Thus, when considering the effects of maternal stress on preterm birth it is important to consider maternal mental health (e.g., depression and anxiety) as a biobehavioral manifestation of stress and thus a key indicator of maternal distress.

### Maternal distress may be underdiagnosed and disproportionately affect patients of Color

In the U.S., only 16 % of Medicaid patients and 9 % with private insurance were screened for perinatal depression in 2021 [90]. Non-Hispanic Black pregnant individuals report higher levels of stress, racism, anxiety, and depression, along with the lowest levels of social support [91]. Black and Hispanic individuals have 10 – 30 % higher rates of antenatal and postpartum depression compared to White individuals [92], [93] yet are significantly less likely to initiate or receive follow-up treatment [94] (reviewed in [68]).

In the U.S., mental health disorders remain under-diagnosed and under-treated at higher rates in communities of Color compared with White communities [95]. Qualitative research in pregnant people of Color reveals consistent themes of feeling “invisible and ignored,” disrespected, negatively stereotyped, and dismissed [96], [97], [98], [99], [100]. The term *obstetric racism* – coined by anthropologist Dána-Ain Davis – emerged to capture the unique discrimination faced by pregnant people of Color within healthcare settings [101]. A recent survey by the Center for Disease Control and Prevention found that one in five pregnant people reported mistreatment during maternity care, and this was most prevalent among people of Color and individuals with public or no insurance [102]. Further, it has been suggested that there are different spontaneous preterm birth phenotypes by different populations in part because stressors are not equitably patterned across populations [103]. Obstetric racism does not appear to be limited to the U.S. Studies from the U.K., Australia, and Canada report similar qualitative data evidencing disparate and poorer treatment for patients of Color within medical settings [104], [105], [106], [107]. Together, these data highlight the importance of qualitative research in understanding health disparities [108].

## The ancient relationship between stress and birth timing

Stress physiology and healthy pregnancy physiology are deeply interwoven [109], reflecting an ancient adaptive strategy to support both maternal and fetal survival in the face of environmental demands. This integration spans multiple biological systems including – immune/inflammatory, cardiovascular, neuroendocrine, and metabolic systems – which coordinate to maintain homeostasis while preparing for the various biological demands of pregnancy [110]. The neuroendocrine system offers a compelling lens to understand the ancient evolutionary roots of the stress-pregnancy connection. By focusing on neuroendocrine signaling, we can trace how mechanisms like the hypothalamic-pituitary-adrenal (HPA) axis have been co-opted to serve both stress response and pregnancy adaptation across species.

### The neuroendocrine stress axis and pregnancy

Neuroendocrine stress hormones play a key role maintaining pregnancy homeostasis and in mediating the endocrine, physiologic, and behavioral response to perceived threat or stress [69]. The neuropeptide corticotropin-releasing hormone (CRH) is a major mediator in the stress response system [69]. Before and during pregnancy, CRH plays a role in the classic stress-response system. CRH stimulates adrenocorticotropin hormone (ACTH) release from the anterior pituitary, which in turn stimulates cortisol production in the adrenal cortex – comprising the HPA axis [111]. CRH is an evolutionarily conserved ancient regulatory molecule found across diverse taxa [112]. During pregnancy, CRH plays a significant regulatory role [112] because the placenta produces 1000 – 10,000 times higher concentrations of CRH compared with non-pregnant states [113]. Placental CRH (pCRH) appears to play a key role in parturition onset [111], [112], [113], [114], [115] and has been described as a “placental clock” due to its exponential rise before imminent labor [116]. pCRH is one of several redundant, overlapping human gestational clocks [117], [118], [119], [120] controlling the timing of birth. CRH is responsive to vascular [121], metabolic [122], and inflammatory signaling [123], all of which have been proposed to mediate the onset of spontaneous preterm birth [18]. CRH has been suggested as a kind of *in utero* rheostat for its role maintaining maternal-fetoplacental symbiosis [124].

Maternal cortisol levels, and premature activation of the fetal HPA axis, have been found to be associated with preterm birth and may indicate concomitantly altered maternal blood CRH levels [31], [117], [125], [126], [127], [128], [129]. Cortisol levels have also been found to be associated with maternal distress such that pregnant people with higher distress had lower rates of change in cortisol across pregnancy [130]. Studies indicate people with major depression in pregnancy may have blunted cortisol awakening response but higher diurnal salivary cortisol [131], [132], [133]. Similarly, other studies have observed lower rates of change in maternal cortisol in people who go on to give birth prematurely [24], [125]. The mixed findings on elevated versus lowered endocrine markers reflects the complex pathophysiology of stress and may indicate differential effects of stress (e.g., hypo- vs. hyper-reactive responses) (Fig. 2) [69], [134],

### Stress-induced developmental plasticity and spontaneous preterm birth

Pregnancy is a highly sensitive period when environmental cues mediated via maternal signaling and physiology can alter gestational duration, fetal growth, and fetal development [57]. Developmental plasticity is a type of phenotypic variation and refers to an organism’s ability to modify morphology or physiology in response to environmental stimuli [59]. Maternal stress is a key environmental cue leading to stress-induced developmental plasticity [36]*.* Stress-induced developmental plasticity describes modifications to growth or development in response to a stressor and is an adaptive reproductive strategy [135] found in mammals, amphibians, reptiles, and birds [136]. A testament to the ancient origins of this strategy is the fact that humans and amphibians not only share similarly conserved reproductive endocrinology, but also a similar capacity for stress-induced developmental plasticity [58], [137], [138], [139]. For example, as tadpoles sense their pond water is drying up they can accelerate metamorphosis, in part, through the neuroendocrine stress axis [140] (reviewed in [36], [68], [141]). Provided they have reached a certain level of maturation, the tadpoles can upregulate CRH and subsequently glucocorticoids to accelerate metamorphosis much the same way we see a precocious rise in CRH in spontaneous preterm birth [140] (reviewed in [36], [68], [141]).

Developmental plasticity during pregnancy, central to the Developmental Origins of Health and Disease (DOHaD) model, suggests that in utero experiences shape fetal development and later disease risk [142]. Stress-induced plasticity may contribute to this process. For instance, war exposure during gestation has been linked to glucocorticoid-related epigenetic changes affecting birth weight [143]. Maternal stress and depression can upregulate FKBP51, enhancing progesterone receptor activity and triggering functional progesterone withdrawal, which may lead to preterm birth [144]. Prenatal stress is also associated with hypermethylation of stress-related genes in offspring, which are linked to depression and blunted cortisol responses [145]. These findings highlight epigenetic modification as a key molecular pathway in developmental plasticity [146], [147].

Understanding stress-induced developmental plasticity provides a biological framework for how maternal stress can influence the timing of birth as an adaptive response. This lens is especially valuable for examining racialized disparities in preterm birth, where chronic exposure to stressors from racism and systemic neglect may act as potent environmental cues influencing birth timing.

### Stress-induced developmental plasticity as a mechanism for racialized disparities in spontaneous preterm birth

Birthing populations facing racism and socio-economic disadvantage experience higher chronic stress, which may help explain their elevated rates of spontaneous preterm birth (reviewed in [36], [68], [141]). A qualitative study found Black individuals perceived stress as a key contributor to preterm birth risk [148]. These patterns likely reflect the multi-level effects of discrimination on health, partly mediated by maternal-fetal-placental neuroendocrine signaling.

**Fig. 3:**
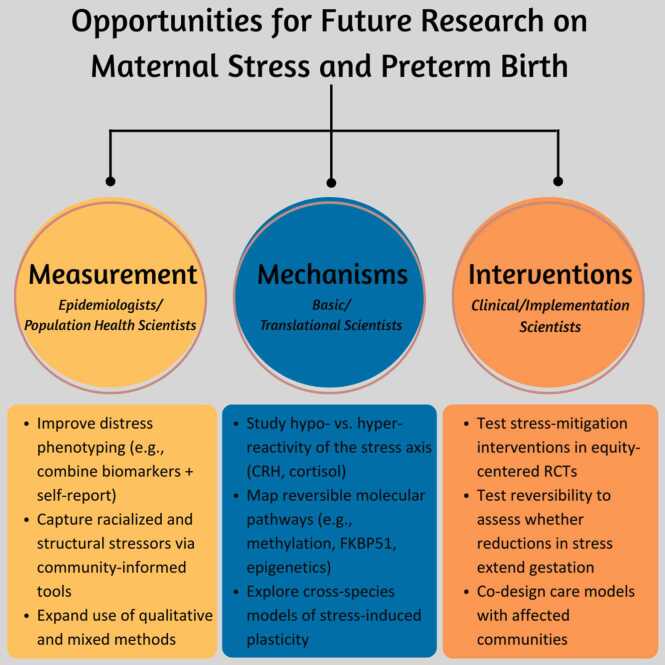
Suggested opportunities for future research on maternal stress and preterm birth based on the evidence reviewed. Opportunities are organized by domain – measurement, mechanisms, and interventions – corresponding to key stakeholder groups: epidemiologists/population health scientists, basic/translational scientists, and clinical scientists.

Differences in maternal stress biomarkers across socially-defined racial groups reflect varying stress exposures [45], [149], [150], [151], [152], [153]. These biomarkers may be elevated or, counterintuitively, blunted in response to stress. Racial/ethnic variations in maternal-placental-fetal hormones have also been linked to preterm birth risk. For example, Black participants had lower overall CRH levels than White participants, but higher mid-pregnancy CRH predicted spontaneous preterm birth before 35 weeks in both groups, with odds ratios of 2.3 (95 % CI 1.1–5.1) for White participants and 5.0 (95 % CI 1.8–13.3) for Black participants[151]. Other studies have observed similar effect modification where groups marginalized by racism demonstrate blunted stress biomarkers [149], [154], while others observed elevated stress biomarkers [155]. Stress biomarkers and pCRH signaling could be altered and more sensitive in individuals experiencing chronic, cumulative stressors from racism. However, more research is needed to determine the effects of hypo- vs. hyper-reactive neuroaxis signaling in pregnancy. Additionally, more high-quality research is needed to assess the nuanced relationships between experiences of racism, plasticity effects, stress biomarkers, and preterm risk (Fig. 3). Viewing spontaneous preterm birth through a framework of stress-induced developmental plasticity reveals two clinically relevant concepts important for maternal pregnancy care: (1) stress effects are incremental and dose-responsive, and (2) the effects of stress may be reversible.

## Clinical implications: stress may be dose-responsive and reversible

### Stress dose-dependently affects gestational length

*In vitro* studies from the 1980s showed that stress-related biological modulators – norepinephrine, acetylcholine, oxytocin, and cortisol – stimulate placental CRH secretion in a dose-dependent manner [156], [157]. Observational studies in humans similarly demonstrate that depression, anxiety, and social disadvantage are associated with shorter gestation in a dose-response fashion [15], [88], [89], [153], [158], [159]. For example, each unit increase in depression score has been linked to a three- to four-day reduction in gestational age [88], while anxiety and social disadvantage also predict gestational shortening of around three days per standard deviation increase [88], [153], [159]. In one large cohort, pregnancy anxiety above the median was associated with nearly double the odds of spontaneous preterm birth (aOR 1.8, 95 % CI 1.3–2.4) [15]. Some studies show maternal depression is linked to significantly shorter gestation – up to eight days less [160] – while childhood trauma predicts steeper pCRH increases during pregnancy [161]. However, not all studies find consistent effects of psychosocial stress on CRH or catecholamines [162]. Experiences of racial discrimination are dose-responsively associated with both depression and increased risk of preterm birth [76], [163], [164], [165], [166]. In summary, these findings underscore the dose-responsive nature of stress on gestational length, with higher levels of depression, anxiety, and social disadvantage consistently linked to shorter pregnancies.

### Developmental plasticity is dynamic and the effects of stress may be reversible

#### Animal studies and environmental enrichment

Returning to the tadpoles discussed earlier, when researchers restored water levels, the tadpoles were able to decelerate maturation in a dose-dependent manner thereby extending metamorphosis to a more optimal time [140]. In this example, we see both the incremental, dose-dependent effects of stress as well as the reversibility of this relationship. In another example, by initiating an enriched environment (EE) protocol, researchers reduced preterm birth by 40 % in an inflammatory mouse model [167]. By placing mice in larger cages with running wheels and objects of differing colors, shapes and textures, researchers observed a reduced corticosterone surge in the EE protocol mice compared with controls after an identical bacterial lipopolysaccharide (LPS) protocol was administered at gestational day 15. Using a similar EE protocol, separate researchers observed reduced anxiety in the mother and increased survivability of the pups upon cesarean delivery at term [168]. In yet another study, researchers were able to reverse the epigenetic effects of prenatal stress on adult offspring through methyl supplementation demonstrating the reversibility of stress at the molecular level [169]. These examples suggest the manifestations of stress may be dampened, mitigated, and even reversed through environment enrichment. Evidence in humans suggests a similar relationship.

#### Human experience of environmental enrichment and preterm birth

As in animal models, enriched positive human experiences in pregnancy during the second trimester associated with increased gestational duration that was dose-dependent, reversible, and reduced the risk of preterm birth [170]. In a separate example, practitioners reduced preterm birth rates through a model of care focused on reducing the unique stressors of discrimination through easy access to respectful, culturally sensitive, patient-centered care [171]. In this model focused on creating a welcoming and inclusive environment where no one is turned away for care, preterm birth for Black birthing individuals was reduced to 8 % compared with a county rate of 13 %, and disparities between Black and White individuals narrowed [171]. Similarly, First Nations women in Brisbane, Australia assigned to a ‘Birthing in Our Community’ care intervention – which provided social, emotional, and cultural support – experienced reduced rates of preterm birth by more than 5 % compared with those that received standard care over a six-year period [172]. In another study, the use of doulas was associated with a 22 % lower odds for preterm birth [173], and in an urban New York City pregnant cohort, doulas were associated with a reduction in preterm birth rates from 12.4 % to 6.3 % [174]. The effects of doulas on preterm birth may be, in part, the result of reductions in maternal perceptions of stress [174], [175], [176], [177], [178], [179], [180], [181]. Community-based initiatives – such as B’More for Healthy Babies, JUST Birth Network, and Roots Community Birth Center – also hold promise for reducing preterm birth and related sequalae including neonatal outcomes and maternal mental health because they cater to the needs of their unique patient population and are highly culturally sensitive [182], [183], [184], [185], [186].

Two recent meta-analyses found that relaxation interventions during pregnancy (e.g., yoga, deep breathing, music) reduced stress, anxiety, and depression and lowered the risk of preterm birth by up to 50 % (RR 0.50, 95 % CI 0.35–0.71) [187], [188]. Stress-reduction approaches, including cognitive behavioral therapy, have been linked to lower cortisol levels, highlighting the connection between stress and pregnancy physiology [189], [190]. A post hoc analysis of a clinical trial found that greater declines in depression during pregnancy, mediated by interpersonal psychotherapy, increased the odds of birth ≥ 39 weeks (OR 1.65, 95 % CI 1.02–2.66) [191].

### Opportunities for identifying, mitigating, and reducing maternal stress in clinical settings

While structural stressors are often outside the control of the provider (e.g., air pollution), there are good tools to screen for maternal distress using validated clinical questionnaires for anxiety, depression, or perceived stress. If maternal stress can be identified and mitigated, it may inform patient care and suggest opportunities to reduce maternal stress and its physiologic consequences.

Guidelines from the American College of Obstetrics and Gynecology (ACOG) encourage clinicians to screen perinatal mental health because treatment could influence the risk for preterm birth [192]. Research demonstrates anxiety and depression measures may also correlate with maternal experiences of racism and discrimination where higher perinatal depression is associated with greater experiences of racial discrimination [166], [193]. However, there are also concerns that cultural differences in symptom presentation (i.e., somatic complaints vs. mood disturbances) may make these screening instruments less effective in individuals of Color [194], [195].

Given the multitude of stressors that are population specific it is difficult to capture one single intervention or study that can resolve and/or summarize this complex relationship. Nonetheless, the evidence reviewed here suggests that while it is outside the reach of providers to change many of the structural stressors facing their patient populations, they can still positively impact birth outcomes by shifting the lens through which they understand maternal stress and spontaneous preterm birth. This shift requires centering the lived experiences of the birthing person, acknowledging broader systems in which that person lives and receives care, and reimagining maternal health as a critical part of reproductive justice.

Interventions to identify, reduce, and mitigate maternal stress include promoting easy access to safe, respectful, culturally sensitive care [171], increasing social support for the birthing person through doulas [173], [174], performing serial mental health screenings across pregnancy [191], [192], and advising patients on stress-reducing interventions and relaxation techniques [187], [188], [191].

## Conclusion

Spontaneous preterm birth is a major contributor to global health inequities. Stress-induced developmental plasticity offers a framework for understanding how social adversity influences birth timing through evolutionarily conserved maternal-fetal-placental signaling. Black feminist theory and reproductive justice frameworks help identify how and where meaningful intervention is possible. *Mapping the margins*—centering the populations most burdened by preterm birth—clarifies where stress is most concentrated and where the greatest potential for impact lies. Prioritizing qualitative research to capture the lived experiences of birthing populations, alongside equity-oriented, community-engaged, and context-specific randomized control trials (RCTs), will deepen our understanding of the nuanced relationship between stress and pregnancy (Fig. 3). Crucially, stress effects are not immutable. Emerging evidence shows that supportive, enriched environments can extend gestation and reduce preterm birth risk. This reversibility presents a critical clinical opportunity to improve patient wellbeing and advance birth equity.

## CRediT authorship contribution statement

**Gabriella Mayne:** Writing – original draft, Conceptualization. **Luwam Ghidei:** Writing – review & editing, Conceptualization. **Ayisha Buckley:** Writing – review & editing. **Wei Perng:** Writing – review & editing. **K. Joseph Hurt:** Writing – review & editing, Conceptualization. **David P. Tracer:** Writing – review & editing, Conceptualization.

## Consent for publication

All authors consent to the publication of this work.

## Funding

The authors have no financial disclosures to report.

## Declaration of Competing Interest

None.
